# Long Non-coding RNA LINC01787 Drives Breast Cancer Progression via Disrupting miR-125b Generation

**DOI:** 10.3389/fonc.2019.01140

**Published:** 2019-11-05

**Authors:** Yongzhen Li, Ying Song, Zhihui Wang, Zheying Zhang, Manman Lu, Yongxia Wang

**Affiliations:** ^1^Department of Pathology, Xinxiang Medical University, Xinxiang, China; ^2^Department of Pathology, The Third Affiliated Hospital of Xinxiang Medical University, Xinxiang, China

**Keywords:** long non-coding RNA, breast cancer, progression, pre-microRNA, microRNA generation

## Abstract

Breast cancer is still the most common and leading cause of cancer-related deaths in women worldwide. Long noncoding RNAs (lncRNAs) and microRNAs (miRNAs) have shown key regulator roles in various cancers. Previous reports have identified miR-125b as a critical tumor suppressor in breast cancer. However, the role of lncRNAs in breast cancer is far from well-characterized. In this study, we identified a novel lncRNA LINC01787, which specifically binds pre-miR-125b, inhibits the binding between DICER and pre-miR-125b, represses the processing of pre-miR-125b by DICER, and therefore induces pre-miR-125b accumulation and represses mature miR-125b generation. Functional assays showed that LINC01787 promotes breast cancer cell proliferation and migration and breast cancer xenograft growth *in vivo*, which is abolished by the mutation of pre-miR-125b binding sites on LINC01787 or overexpression of miR-125b. Furthermore, LINC01787 is up-regulated in breast cancer tissues and is associated with advanced stages and poor survival. The expression of LINC01787 is inversely associated with that of miR-125b in breast cancer tissues. In conclusion, our findings identified a novel up-regulated and oncogenic lncRNA LINC01787 in breast cancer, which binds pre-miR-125b and represses mature miR-125b generation. Our data suggests LINC01787 as a potential prognostic biomarker and therapeutic target for breast cancer.

## Introduction

According to global cancer statistics in 2018, breast cancer remains the most commonly diagnosed cancer and the leading cause of cancer death in women worldwide (1). Breast cancer accounts for 24.2% of the total incidence of cancers in women and 15.0% of the total deaths of cancer in women, with 2,088,849 estimated new cases and 358,989 estimated deaths in 2018 worldwide (1). Despite advances in surgery, chemotherapy, radiotherapy, endocrine therapy, molecular-targeted therapy, and immunotherapy, breast cancer remains the cause of a vast number of deaths (2–5). Currently, the outcome of breast cancer patients is still far from satisfactory, which is largely due to an unclear understanding of the molecular mechanisms underlying the initiation and progression of breast cancer (6–9).

Whole-genome and whole-transcriptome sequencings have found that most human genomes are transcribed, and while only about 2% of human genomes encode proteins, which suggests that most of human transcriptomes are non-coding RNAs (10). Among these great number of non-coding RNAs, microRNA (miRNA) and long non-coding RNA (lncRNA) are two classes of regulatory RNAs, that play important roles in various physiological and pathological processes (11–14). miRNAs are small non-coding RNAs with 19–25 nucleotides in length (15, 16). miRNAs regulate gene expression mainly via binding the 3′-untranslated region (3′-UTR) of target mRNAs, inducing target mRNAs degradation and/or repressing target mRNAs translation, and lastly inhibiting target genes expression (17–19). Therefore, miRNAs may have oncogenic or tumor-suppressive roles via targeting tumor suppressors or oncogenes (20–22). In our previous study, we have found that miR-125b exerts tumor suppressive roles in breast cancer via targeting KIAA1522 (23). The tumor suppressive roles of miR-125b in breast cancer were also verified in other reports with several new miR-125b targets being identified, including ETS and SNAI1 (24, 25).

lncRNAs are long non-coding RNAs with more than 200 nucleotides that are long and lack an extended open reading frame (26). Whole-transcriptome sequencings have found that the human transcriptome contains more than 58,000 lncRNAs, while the number of protein-coding genes is only about 21,000 (10). Although the expression of lncRNAs is relatively lower than that of protein-coding genes, the expression of lncRNAs are more disease and tissue-specific (27). Aberrant expression of lncRNAs are frequently observed in various cancers, including breast cancer (28). Like miRNAs, lncRNAs play oncogenic or tumor-suppressive roles in cancers (29–31). lncRNAs may modulate the proliferation, apoptosis, cell cycle, migration, invasion, growth, metastasis, drug-resistance, angiogenesis, and so on of cancer cells (32–35). Compared with miRNAs, the mechanisms of action of lncRNAs are relatively more complex and varied (36). lncRNAs can modulate protein-coding genes′ expression at a transcriptional level, post-transcriptional level, translational level, and post-translational modification (37–39). Furthermore, lncRNAs can modulate miRNAs and further regulate the expression of key miRNAs targets (40). Routinely, lncRNAs can sponge miRNAs and relieve the repressing roles of miRNAs on their targets (41). Furthermore, lncRNAs may regulate the generation of miRNAs via binding pre-miRNAs (42). miRNA-coding genes first transcribe into pri-miRNAs, which are further processed by Drosha to generate pre-miRNAs. Then, the pre-miRNAs are translocated to the cytoplasm and processed by DICER to generate mature miRNAs. We have found that miR-125b is a critical tumor suppressor in breast cancer (23). We further hypothesized that lncRNAs, which can regulate miR-125b, may also have important roles in breast cancer.

First, we predicted the lncRNAs which could specifically bind miR-125b using starBase (http://starbase.sysu.edu.cn/). However, none of these predicted lncRNAs show a significant correlation with the survival of breast cancer patients in The Cancer Genome Atlas (TCGA) data. Next, we searched for lncRNAs that may modulate miR-125b generation. The lncRNAs that could bind pre-miR-125b were searched for using Blast (https://blast.ncbi.nlm.nih.gov/Blast.cgi). Only lncRNA LINC01787 was predicted to have a strong binding potential with pre-miR-125b. Furthermore, analyzing the TCGA data found that a high expression of LINC01787 is correlated with poor survival of breast cancer patients with a hazard ratio of 1.52. In this study, we further investigated the expression, clinical association, roles, and functional mechanisms of LINC01787 in breast cancer.

## Materials and Methods

### Cell Culture

Human breast cancer cell lines MDA-MB-231 and MCF-7 were acquired from the American Type Culture Collection (Manassas, VA, USA) and passaged in our lab. The cells were cultured in DMEM medium (Invitrogen, Thermo Fisher Scientific, Carlsbad, CA, USA) supplemented with 10% fetal bovine serum (Invitrogen, Thermo Fisher Scientific) and 100 U/ml penicillin and 100 mg/ml streptomycin (Invitrogen, Thermo Fisher Scientific). The cells were incubated in a humidified incubator containing 5% CO_2_ at 37°C.

### Plasmids Construction and Transfection

LINC01787 full-length sequences were PCR-amplified from human cDNA using the Thermo Scientific Phusion Flash High-Fidelity PCR Master Mix (Thermo Fisher Scientific) with the primers 5′-CCCAAGCTTGAGAATTACTCTGGATTGTAAGC-3′ (forward) and 5′-GCTCTAGAGGAACCAAAATGGTCCAGGAT-3′ (reverse). The PCR products were then cloned into the Hind III and Xba I site of pcDNA™3.1(+) plasmid (Invitrogen, Thermo Fisher Scientific) and pSPT19 plasmid (Roche, Mannheim, Germany) to generate pcDNA3.1-LINC01787 and pSPT19-LINC01787, respectively. LINC01787 full-length sequences with pre-miR-125b binding sites mutated were synthesized by GenScript (Nanjing, China) and cloned into the Hind III and Xba I site of pcDNA™3.1(+) and pSPT19 plasmid to generate pcDNA3.1-LINC01787-mut and pSPT19-LINC01787-mut, respectively. Two independent cDNA oligonucleotides targeting LINC01787 (shLINC01787-1 and shLINC01787-2) were synthesized by GenePharma (Shanghai, China) and cloned into the shRNA expression vector pGPH1/Neo (GenePharma). The target site of shLINC01787-1 was 5′-GCTGATAAAGACATACCTAAG-3′. The target site of shLINC01787-2 was 5′-GCTTCTGTTGGCTAGCAATAA-3′. 3′-UTR of KIAA1522, ETS1, and SNAI1 containing miR-125b targeting sites were PCR-amplified from human cDNA using the Thermo Scientific Phusion Flash High-Fidelity PCR Master Mix (Thermo Fisher Scientific) with the primers 5′-CGAGCTCCTGGCGGAGAATGGAGGTGT-3′ (forward) and 5′-GCTCTAGAGGGTGGTTGGTGAGTTGAGG-3′ (reverse) for KIAA1522, 5′-CGAGCTCGAGACCTTCCAAGGACAG-3′ (forward) and 5′-GCTCTAGAGCAAGCAATAATTGATACCC-3′ (reverse) for ETS1, 5′-CGAGCTCCTCCCTCTTCCTCTCCATAC-3′ (forward) and 5′-GCTCTAGACCATTACTCACAGTCCCTTTTC-3′ (reverse) for SNAI1. The PCR products were then cloned into the Sac I and Xba I site of pmirGLO Dual-Luciferase miRNA Target Expression Vector (Promega, Madison, WI, USA) to generate pmirGLO-KIAA1522, pmirGLO-ETS1, and pmirGLO-SNAI1, respectively. Plasmids transfection was carried out using Lipofectamine™ 3000 (Invitrogen, Thermo Fisher Scientific) following the provided protocol.

### RNA Extraction and Quantitative Real-Time PCR (qRT-PCR)

Total RNA was extracted from cultured cells and indicated tissues using Trizol reagent (Invitrogen, Thermo Fisher Scientific) following the provided protocol. After quantification using NanoDrop-1000 (Thermo Fisher Scientific), the extracted RNA was used to synthesize the first strand cDNA with BeyoRT™ cDNA synthesis kit (Beyotime, Haimen, Jiangsu, China). Quantitative real-time PCR (qRT-PCR) was performed on an ABI 7500 Real-time PCR system (Applied Biosystems, Thermo Fisher Scientific) using BeyoFast™ SYBR Green qPCR Mix (Beyotime) with the primers 5′-AAGCAGAAAGCAAGAGTG-3′ (forward) and 5′-CCGTTGTATGTATGTACCA-3′ (reverse) for LINC01787, 5′-AACGGATTTGGTCGTATTG-3′ (forward) and 5′-GGAAGATGGTGATGGGATT-3′ (reverse) for GAPDH, 5′-GGGAAATCGTGCGTGACATTAAG-3′ (forward) and 5′-TGTGTTGGCGTACAGGTCTTTG-3′ (reverse) for β-actin, 5′-GCTTCGGCAGCACATATACTAAAAT-3′ (forward) and 5′-CGCTTCACGAATTTGCGTGTCAT-3′ (reverse) for U6. GAPDH was used as endogenous control for the calculation of LINC01787 expression. For the quantitation of pre-miR-125b-1, pre-miR-125b-2, pre-miR-483, and miR-125b expression, qRT-PCR was carried out as above using Applied Biosystems™ TaqMan™ pre-miRNA Gene Expression Assay (Applied Biosystems, Thermo Fisher Scientific) and TaqMan™ MicroRNA Assay (Applied Biosystems, Thermo Fisher Scientific), respectively. The results were calculated using the 2^−ΔΔCt^ method. To quantify the exact molecular numbers of LINC01787, pre-miR-125b-1, and pre-miR-125b-2 per cell, standard curves were formulated with limit dilution approaches using LINC01787 expressing vector pcDNA3.1-LINC01787, and pre-miR-125b-1 and pre-miR-125b-2 expressing vectors purchased from GenePharma as standard templates. The exact molecular numbers of LINC01787, pre-miR-125b-1, and pre-miR-125b-2 per cell were calculated according to cell counts and molecular weights.

### Isolation of Cytoplasmic and Nuclear RNA

Cytoplasmic and nuclear RNA were extracted from indicated breast cancer cells with the Cytoplasmic & Nuclear RNA Purification Kit (Norgen, Belmont, CA) following the provided protocol. The RNA present in the cytoplasm and nucleus was measured by qRT-PCR as above.

### RNA Pull-Down Assay

Wild type LINC01787 and pre-miR-125b binding sites, mutated LINC01787 were *in vitro* transcribed and biotin-labeled from pSPT19-LINC01787 and pSPT19-LINC01787-mut, respectively, using the Biotin RNA Labeling Mix (Roche) and T7 RNA polymerase (Roche). After treatment with DNase I (Roche), the *in vitro* transcribed biotin-labeled LINC01787-wt and LINC01787-mut were purified with the RNeasy Mini Kit (Qiagen, Valencia, CA, USA). Then, 3 μg of purified biotin-labeled LINC01787-wt and LINC01787-mut were incubated with 1 mg of whole-cell lysates from MDA-MB-231 cells at 25°C for 1 h. The complexes were enriched using the streptavidin agarose beads (Invitrogen, Thermo Fisher Scientific). The RNA present in the pull-down material was measured by qRT-PCR as above. In addition, the binding between RNA and RNA was verified using LINC01787 antisense biotinylated probes and the EZ- Magna ChIRP RNA Interactome Kit (Millipore, Bedford, MA, USA) following the provided protocol. The sequences of LINC01787 antisense probes were: 1, 5′-atttgcttacaatccagagt-3′; 2, 5′-gaggcaataggctttcaagt-3′; 3, 5′-tgcttatcgttttgcttcat-3′; 4, 5′-gccaattctcattgaactgt-3′; 5, 5′-tagttgttgcttgtaacctc-3′; 6, 5′-tgggtcagattttctttacc-3′; 7, 5′-caattggaagccatactggt-3′; 8, 5′-caaaatggtccaggatgctc-3′.

### RNA Immunoprecipitation (RIP) Assay

pcDNA3.1, pcDNA3.1-LINC01787, pcDNA3.1-LINC01787-mut, shCtl, shLINC01787-1, or shLINC01787-2 was transfected into MDA-MB-231 cells. Forty-eight hours after transfection, these cells were used to carry put RNA immunoprecipitation (RIP) assays with the Magna RIP RNA-Binding Protein Immunoprecipitation Kit (Millipore) and a DICER specific antibody (5 μg per reaction; ab14601, Abcam, Cambridge, MA, USA) following the provided protocol.

### Luciferase Reporter Assay

pmirGLO, pmirGLO-KIAA1522, pmirGLO-ETS1, or pmirGLO-SNAI1 was co-transfected with pcDNA3.1, pcDNA3.1-LINC01787, pcDNA3.1-LINC01787-mut into MCF-7 cells. pmirGLO, pmirGLO-KIAA1522, pmirGLO-ETS1, or pmirGLO-SNAI1 was co-transfected with shCtl, shLINC01787-1, or shLINC01787-2 into MDA-MB-231 cells. Forty-eight hours after transfection, the firefly luciferase activity was detected with the Dual-Luciferase Reporter Assay System (Promega) and normalized to Renilla luciferase activity.

### Western Blot

Total protein was extracted from indicated cultured cells with RIPA lysis buffer (Beyotime) added to a protease inhibitor PMSF (Beyotime). The concentrations of extracted proteins were detected using Enhanced BCA Protein Assay Kit (Beyotime). Equal amount of protein was separated by sodium dodecyl sulfate-polyacrylamide gel electrophoresis (SDS-PAGE). Next, the separated proteins were transferred to polyvinylidene fluoride (PVDF) membrane (Beyotime). After blocking using fat free milk, the membranes were incubated with primary antibodies against KIAA1522 (ab122203, 1:500, Abcam), ETS1 (ab220361, 1:1,000, Abcam), SNAI1 (#3879, 1:1,000, Cell Signaling Technology, Boston, USA), or GAPDH (ab8245, 1:10,000, Abcam) overnight at 4°C. After being washed using TBST three times, the membranes were further incubated with Goat anti-Rabbit IgG H&L (IRDye^®^ 800CW) preadsorbed (ab216773, 1:10,000, Abcam) or Goat anti-Mouse IgG H&L (IRDye^®^ 680RD) preadsorbed (ab216776, 1:10,000, Abcam) for 1 h at room temperature and then imaged using the Odyssey infrared scanner (Li-Cor, Lincoln, NE, USA).

### Stable Cell Lines Construction

To construct wild type LINC01787 (LINC01787-wt) or pre-miR-125b binding sites mutated LINC01787 (LINC01787-mut) stably overexpressed breast cancer cells, pcDNA3.1, pcDNA3.1-LINC01787, pcDNA3.1-LINC01787-mut was transfected into MDA-MB-231 and MCF-7 cells. Forty-eight hours after transfection, the cells were treated with neomycin to select LINC01787 stably overexpressed cells. To construct LINC01787 stably depleted breast cancer cells, shCtl, shLINC01787-1, or shLINC01787-2 were transfected into MDA-MB-231 and MCF-7 cells. Forty-eight hours after transfection, the cells were treated with neomycin to select LINC01787 stably depleted cells. To construct miR-125b and LINC01787 concurrently stably overexpressed breast cancer cells, miR-125b overexpression lentivirus (#HmiR0178-MR04, FulenGen, Guangzhou, China) was infected into LINC01787 stably overexpressed MDA-MB-231 cells. Four days after infection, the cells were treated with neomycin and puromycin to select miR-125b and LINC01787 concurrently stably overexpressed cells. Overexpression efficiencies were confirmed by qRT-PCR as above.

### Cell Proliferation Assay

A cell counting kit-8 (CCK-8) assay and a 5-ethynyl-2'-deoxyuridine (EdU) incorporation assay were undertaken to analyze cell proliferation. For the CCK-8 assay, indicated breast cancer cells were seeded 3,000 cells per well into 96-well plates and incubated for 0–3 days. At an indicated time, the CCK-8 reagent (Beyotime) was added to the plates and the cells were further incubated for 2 h. The optical density at 450 nm was detected to calculate cell proliferation. EdU incorporation assay was undertaken using the EdU Kit (RiboBio, Guangzhou, China) following the provided protocol. The results were acquired using Zeiss photomicroscope (Carl Zeiss, Oberkochen, Germany) and analyzed using Image-Pro plus 6.0 software.

### Cell Migration Assay

A Transwell migration assay was undertaken to analyze cell migration. Indicated breast cancer cells re-suspended in fetal bovine serum free DMEM were seeded to the upper chamber of a transwell insert (Millipore). DMEM with 10% fetal bovine serum was added to the lower chamber. After incubation for 48 h, the cells remaining on the upper chamber were wiped off with a cotton swab. The cells migrated into the lower surface were fixed using 4% paraformaldehyde, stained using 0.1% crystal violet, and photographed using Zeiss photomicroscope.

### Animal Experiment

Four-six week old female athymic BALB/c nude mice were acquired from Shanghai Lingchang Biological Technology Ltd (Shanghai, China). 3 × 10^6^ indicated breast cancer cells were subcutaneously implanted into the mice. Subcutaneous xenograft volume was measured weekly using a caliper and calculated following the formula V = 0.5 × L × S^2^ (L, longest diameter; S, shortest diameter). At the 28th day after implantation, the mice were sacrificed, and subcutaneous xenografts were resected and weighed. The subcutaneous xenografts were fixed in formalin and made into paraffin-embedded sections. The paraffin-embedded sections were stained with primary antibodies against PCNA (ab29, 1:6,000, Abcam), KIAA1522 (ab122203, 1:150, Abcam), ETS1 (ab220361, 1:500, Abcam), or SNAI1 (ab53519, 1:1,000, Abcam) following the routine immunohistochemistry (IHC) method. The sections were also used to perform terminal deoxynucleotidyl transferase (TdT)-mediated dUTP nick end labeling (TUNEL) assay with the *in situ* Cell Death Detection Kit (Roach) following the provided protocol. The animal experiments were reviewed and approved by the Ethics Committee of Xinxiang Medical University (Xinxiang, China).

### Clinical Tissues

Eighty-nine pairs of breast cancer tissues and normal adjacent tissues were obtained from breast cancer patients who received surgery at Xinxiang Medical University (Xinxiang, China). All tissues were diagnosed by pathological examination and stored at −80°C until use. The use of clinical tissues was reviewed and approved by the Ethics Committee of Xinxiang Medical University (Xinxiang, China). Written informed consents were acquired from all patients.

### Statistical Analysis

Statistical analyses were undertaken using GraphPad Prism v6.0 (GraphPad Software, La Jolla, CA, USA). For comparison, one-way ANOVA followed by Dunnett's multiple comparisons test, Kruskal-Wallis test followed by Dunn's multiple comparisons test, Wilcoxon signed rank test, Spearman correlation analysis, Log-rank test, and Pearson chi-square test were performed as indicated. Difference was considered as significant when *P* < 0.05.

## Results

### LINC01787 Binds pre-miR-125b and Represses miR-125b Generation

**Figure 1:**
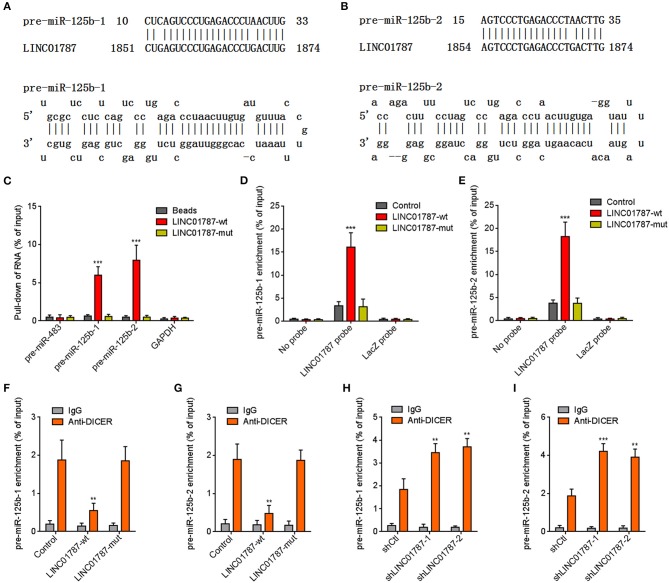
LINC01787 binds pre-miR-125b and represses the binding between pre-miR-125b and DICER. **(A,B)** Schematic diagram of the predicted interaction between LINC01787 and pre-miR-125b. **(C)** RNA pull-down assays using *in vitro* transcribed biotin-labeled LINC01787 was performed to detect the binding between LINC01787 and pre-miR-125b. **(D,E)** After transient overexpression of wild type LINC01787 (LINC01787-wt) or pre-miR-125b binding sites mutated LINC01787 (LINC01787-mut) in MDA-MB-231 cells, RNA pull-down assays using LINC01787 antisense biotinylated probes were performed to detect the binding between LINC01787 and pre-miR-125b-1 **(D)** or pre-miR-125b-2 **(E)**. **(F,G)** After transient overexpression of LINC01787-wt or LINC01787-mut in MDA-MB-231 cells, RIP assays were performed to detect the binding between DICER and pre-miR-125b-1 **(F)** or pre-miR-125b-2 **(G)**. **(H,I)** After transient transfection of LINC01787 specific shRNAs into MDA-MB-231 cells, RIP assays were performed to detect the binding between DICER and pre-miR-125b-1 **(H)** or pre-miR-125b-2 **(I)**. Results are shown as mean ± SD of 3 independent experiments. ^**^P < 0.01, ^***^P < 0.001 by one-way ANOVA followed by Dunnett's multiple comparisons test, compared with beads, control, or shCtl group.

**Figure 2:**
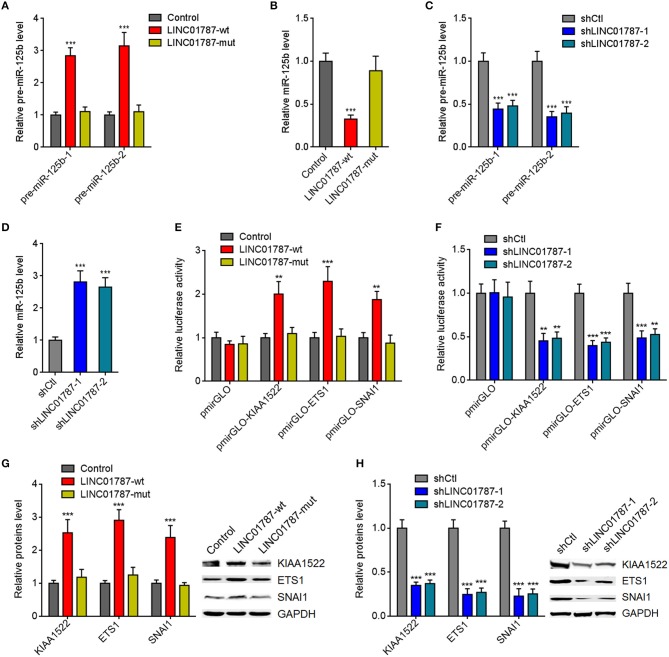
LINC01787 represses miR-125b generation and up-regulates the expression of miR-125b targets. **(A)** After transient overexpression of wild type LINC01787 (LINC01787-wt) or pre-miR-125b binding sites mutated LINC01787 (LINC01787-mut) in MDA-MB-231 cells, pre-miR-125b levels were measured by qRT-PCR. **(B)** After transient overexpression of LINC01787-wt or LINC01787-mut in MDA-MB-231 cells, miR-125b levels were measured by qRT-PCR. **(C)** After transient silencing of LINC01787 in MDA-MB-231 cells, pre-miR-125b levels were measured by qRT-PCR. **(D)** After transient silencing of LINC01787 in MDA-MB-231 cells, miR-125b levels were measured by qRT-PCR. **(E)** Luciferase activity in MCF-7 cells co-transfected with luciferase reporters containing nothing, 3′-UTR of KIAA1522, 3′-UTR of ETS1, or 3′-UTR of SNAI1 and LINC01787-wt or LINC01787-mut overexpression plasmids. Results are shown as the relative ratio of firefly luciferase activity to renilla luciferase activity. **(F)** Luciferase activity in MDA-MB-231 cells co-transfected with luciferase reporters containing nothing, 3′-UTR of KIAA1522, 3′-UTR of ETS1, or 3′-UTR of SNAI1 and LINC01787 specific shRNAs. Results are shown as the relative ratio of firefly luciferase activity to renilla luciferase activity. **(G)** After transient overexpression of LINC01787-wt or LINC01787-mut in MCF-7 cells, the expression of miR-125b targets KIAA1522, ETS1, and SNAI1 was measured by western blot. **(H)** After transient silencing of LINC01787 in MDA-MB-231 cells, the expression of miR-125b targets KIAA1522, ETS1, and SNAI1 was measured by western blot. Results are shown as mean ± SD of 3 independent experiments. ^**^P < 0.01, ^***^P < 0.001 by one-way ANOVA followed by Dunnett's multiple comparisons test, compared with control or shCtl group.

The interaction between LINC01787 and pre-miR-125b-1 and pre-miR-125b-2 was predicted by Blast (https://blast.ncbi.nlm.nih.gov/Blast.cgi) (Figures 1A,B). The nearly completely consistent sequences between LINC01787 and stem regions of both pre-miR-125b implied that LINC01787 may bind another stranded of stem regions of both pre-miR-125b (Figures 1A,B). Moreover, both pre-miR-125b and LINC01787 were mainly located in the cytoplasm in breast cancer cells (Figures S1A,B), which support the potential interaction between LINC01787 and pre-miR-125b. To investigate whether LINC01787 could bind pre-miR-125b, RNA pull-down assays were undertaken using *in vitro*-transcribed biotin-labeled LINC01787. The results showed that both pre-miR-125b was significantly enriched in the pull-down material acquired by biotin-labeled LINC01787 (Figure 1C, Figure S1C). Mutation of the pre-miR-125b binding sites on LINC01787 abolished the specific enrichment of pre-miR-125b (Figure 1C, Figure S1C). After transient overexpression of wild-type (wt) or pre-miR-125b binding sites-mutated LINC01787 in MDA-MB-231 cells via transfection of LINC01787-wt or LINC01787-mut overexpression plasmids (Figure S1D), LINC01787 antisense biotinylated probes was used to pulldown LINC01787 and interacted RNAs. The results revealed that both pre-miR-125b-1 and pre-miR-125b-2 were significantly pulled down by LINC01787-wt, which was abolished by the mutation of pre-miR-125b binding sites (Figures 1D,E). Pre-miRNAs are bound and processed by DICER to generate mature miRNAs. Therefore, we further explored whether the direct binding between LINC01787 and pre-miR-125b disturb the binding and process of pre-miR-125b by DICER. After transient overexpression of LINC01787-wt or LINC01787-mut in MDA-MB-231 cells, RIP assays were performed to detect the binding between pre-miR-125b and DICER. The results revealed that DICER could bind both pre-miR-125b (Figures 1F,G). LINC01787 overexpression markedly inhibited the binding between DICER and both pre-miR-125b, which was abolished by the mutation of pre-miR-125b binding sites on LINC01787 (Figures 1F,G). Conversely, LINC01787 knockdown markedly promoted the binding between DICER and both pre-miR-125b (Figures 1H,I, Figure S1E). The previously reported binding between pre-miR-483 and DICER was used to verify RIP efficiencies, which was not regulated by LINC01787 (Figures S1F,G). The exact number of molecules of LINC01787, pre-miR-125b-1, and pre-miR-125b-2 were quantified in MDA-MB-231 cells. The results showed that the number of molecules of LINC01787 was comparable with that of pre-miR-125b-1 and pre-miR-125b-2 in breast cancer cells (Figure S1H), which support the effects of LINC01787 on pre-miR-125b via direct interaction. Next, we measured pre-miR-125b and mature miR-125b expression levels in MDA-MB-231 cells after transient overexpression or knockdown of LINC01787. The results revealed that LINC01787 overexpression promoted the accumulation of both pre-miR-125b and reduced mature miR-125b level, which were abolished by the mutation of pre-miR-125b binding sites on LINC01787 (Figures 2A,B). LINC01787 knockdown reduced both pre-miR-125b levels and increased mature miR-125b level (Figures 2C,D). Therefore, these data suggested that LINC01787 binds pre-miR-125b, represses the binding and cleavage of pre-miR-125b by DICER, and therefore inhibits the generation of mature miR-125b.

miR-125b is reported to play tumor suppressive roles via targeting KIAA1522, ETS1, and SNAI1 in breast cancer (23–25). We further investigated the effects of LINC01787 on miR-125b targets KIAA1522, ETS1, and SNAI1. 3′-UTR of KIAA1522, ETS1, or SNAI1, which contain miR-125b interaction sites, were cloned into a reporter vector downstream of the firefly luciferase gene. Luciferase reporter assays showed that LINC01787 overexpression increased the luciferase activities of the reporters containing 3′-UTR of KIAA1522, ETS1, or SNAI1, which were abolished by the mutation of pre-miR-125b binding sites on LINC01787 (Figure 2E, Figure S1I). LINC01787 knockdown reduced the luciferase activities of the reporters containing 3′-UTR of KIAA1522, ETS1, or SNAI1 (Figure 2F, Figure S1J). Western blot results showed that LINC01787 overexpression up-regulated the protein levels of KIAA1522, ETS1, and SNAI1, which were abolished by the mutation of pre-miR-125b binding sites on LINC01787 (Figure 2G, Figures S1K,L). LINC01787 knockdown reduced the protein levels of KIAA1522, ETS1, and SNAI1 (Figure 2H, Figures S1M,N). These data suggested that LINC01787 up-regulates the expression of miR-125b targets in a miR-125b dependent manner.

### LINC01787 Overexpression Promotes Breast Cancer Cell Proliferation and Migration in a miR-125b Dependent Manner

**Figure 3:**
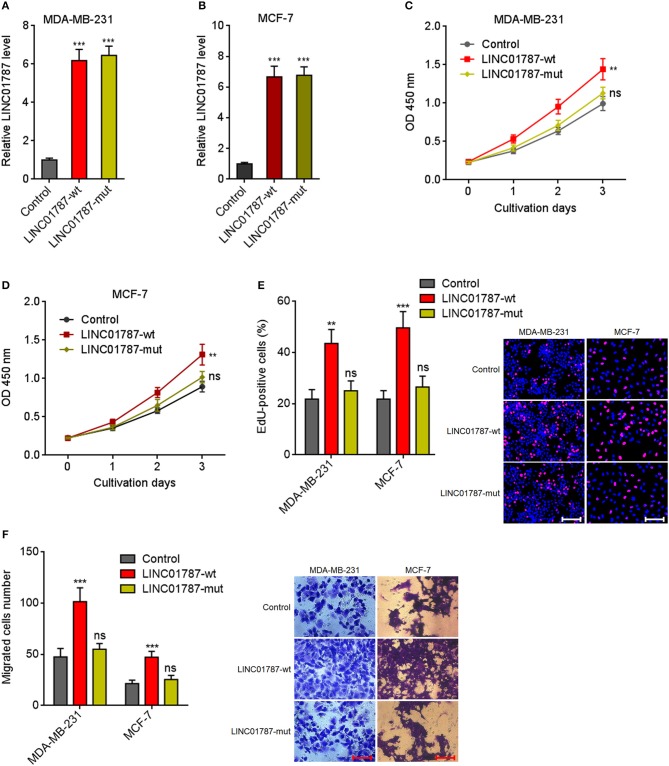
LINC01787 overexpression promotes breast cancer cell proliferation and migration. **(A,B)** LINC01787 expression in wild type LINC01787 (LINC01787-wt) or pre-miR-125b binding sites mutated LINC01787 (LINC01787-mut) stably overexpressed MDA-MB-231 **(A)** or MCF-7 **(B)** cells was detected by qRT-PCR. **(C,D)** Cell proliferation of LINC01787-wt or LINC01787-mut stably overexpressed MDA-MB-231 **(C)** or MCF-7 **(D)** cells was detected by CCK-8 assays. **(E)** Cell proliferation of LINC01787-wt or LINC01787-mut stably overexpressed MDA-MB-231 and MCF-7 cells was detected by EdU incorporation assays. Red colors indicate EdU-positive and proliferative cells. Scale bar = 100 μm. **(F)** Cell migration of LINC01787-wt or LINC01787-mut stably overexpressed MDA-MB-231 and MCF-7 cells was detected by transwell assays. Scale bar = 100 μm. Results are shown as mean ± SD of 3 independent experiments. ^**^P < 0.01, ^***^P < 0.001, ns, not significant by one-way ANOVA followed by Dunnett's multiple comparisons test, compared with control group.

Due to LINC01787 repressing miR-125b generation and miR-125b has tumor suppressive roles in breast cancer, we next investigated the roles of LINC01787 in breast cancer. Wild-type (wt) or pre-miR-125b binding sites mutated LINC01787 stably overexpressed MDA-MB-231 and MCF7 cells were constructed via stable transfection of LINC01787-wt or LINC01787-mut overexpression plasmids (Figures 3A,B). CCK-8 assays showed that LINC01787 overexpression significantly accelerated breast cancer cell proliferation, which was abolished by the mutation of pre-miR-125b binding sites (Figures 3C,D). EdU incorporation assays also revealed that LINC01787 overexpression promoted breast cancer cell proliferation, which was abolished by the mutation of pre-miR-125b binding sites (Figure 3E). Transwell assays showed that LINC01787 overexpression markedly increased migration ability of breast cancer cells, which was abolished by the mutation of pre-miR-125b binding sites (Figure 3F). These data suggested that LINC01787 overexpression promotes breast cancer cell proliferation and migration in a miR-125b dependent manner.

### LINC01787 Knockdown Inhibits Breast Cancer Cell Proliferation and Migration

**Figure 4:**
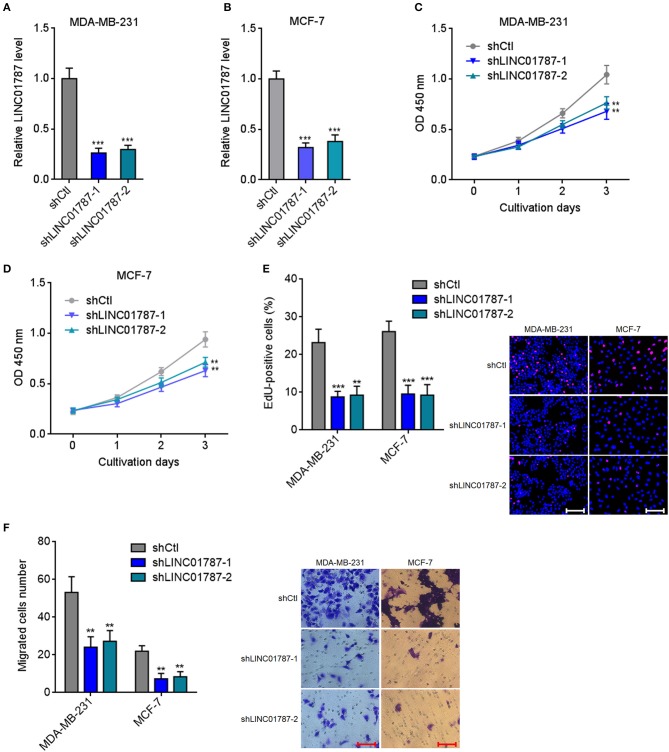
LINC01787 knockdown inhibits breast cancer cell proliferation and migration. **(A,B)** LINC01787 expression in LINC01787 stably depleted MDA-MB-231 **(A)** or MCF-7 **(B)** cells was detected by qRT-PCR. **(C,D)** Cell proliferation of LINC01787 stably depleted MDA-MB-231 **(C)** or MCF-7 **(D)** cells was detected by CCK-8 assays. **(E)** Cell proliferation of LINC01787 stably depleted MDA-MB-231 and MCF-7 cells was detected by EdU incorporation assays. Red colors indicate EdU-positive and proliferative cells. Scale bar = 100 μm. **(F)** Cell migration of LINC01787 stably depleted MDA-MB-231 and MCF-7 cells was detected by transwell assays. Scale bar = 100 μm. Results are shown as mean ± SD of 3 independent experiments. ^**^P < 0.01, ^***^P < 0.001 by one-way ANOVA followed by Dunnett's multiple comparisons test, compared with shCtl group.

To completely elucidate the roles of LINC01787 in breast cancer, LINC01787 stably depleted MDA-MB-231 and MCF7 cells were constructed via stable transfection of two independent LINC01787 specific shRNAs (Figures 4A,B). CCK-8 assays showed that LINC01787 knockdown significantly repressed breast cancer cell proliferation (Figures 4C,D). EdU incorporation assays also revealed that LINC01787 knockdown reduced breast cancer cell proliferation (Figure 4E). Transwell assays showed that LINC01787 knockdown markedly decreased migration ability of breast cancer cells (Figure 4F). These data suggested that LINC01787 knockdown inhibits breast cancer cell proliferation and migration.

### LINC01787 Promotes Breast Cancer Xenograft Growth in a miR-125b Dependent Manner

**Figure 5:**
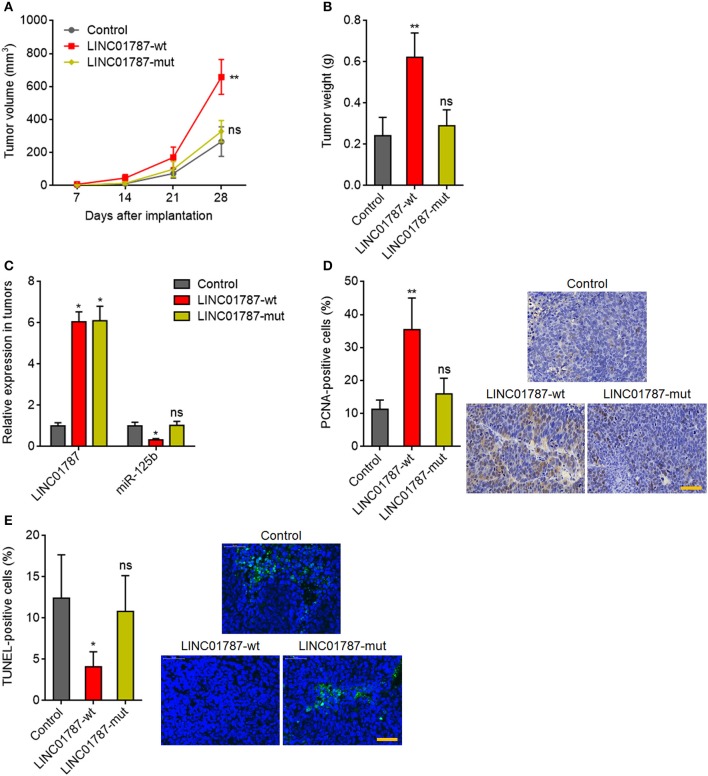
LINC01787 promotes breast cancer xenograft growth *in vivo*. **(A)** Wild type LINC01787 (LINC01787-wt) or pre-miR-125b binding sites mutated LINC01787 (LINC01787-mut) stably overexpressed MDA-MB-231 cells were subcutaneously implanted into nude mice. Tumor volume was detected every 7 days. **(B)** Subcutaneous tumor weight was detected at the 28th day after implantation. **(C)** LINC01787 and miR-125b expression in xenograft from **(B)** was detected by qRT-PCR. **(D)** *In vivo* cell proliferation of LINC01787-wt or LINC01787-mut stably overexpressed MDA-MB-231 cells was detected by PCNA IHC staining. Scale bar = 50 μm. **(E)** *In vivo* cell apoptosis of LINC01787-wt or LINC01787-mut stably overexpressed MDA-MB-231 cells was detected by TUNEL staining. Scale bar = 50 μm. Results are shown as mean ± SD of 5 mice in each group. ^*^P < 0.05, ^**^P < 0.01, ns, not significant by Kruskal–Wallis test followed by Dunn's multiple comparisons test, compared with control group.

Next, we investigated the effects of LINC01787 in breast cancer *in vivo*. LINC01787-wt or LINC01787-mut stably overexpressed MDA-MB-231 cells were subcutaneously implanted into nude mice. The results showed that LINC01787 overexpression significantly promoted MDA-MB-231 xenograft growth *in vivo*, which was abolished by the mutation of pre-miR-125b bind sites (Figures 5A,B). LINC01787 overexpression efficiencies and the repressive roles of LINC01787 on miR-125b were further confirmed in the xenograft (Figure 5C). Proliferation marker PCNA IHC staining showed that the xenograft formed by LINC01787 overexpressed MDA-MB-231 cells had significantly more PCNA positive cells than that formed by control cells (Figure 5D). The increasing of PCNA positive cells was abolished by the mutation of pre-miR-125b bind sites (Figure 5D). TUNEL assays showed that the xenograft formed by LINC01787 overexpressed MDA-MB-231 cells had significantly less apoptotic cells than that formed by control cells, which was also abolished by the mutation of pre-miR-125b bind sites (Figure 5E). These data suggested that LINC01787 promotes breast cancer xenograft growth *in vivo* in a miR-125b dependent manner.

### miR-125b Overexpression Reverses the Oncogenic Roles of LINC01787 in Breast Cancer

**Figure 6:**
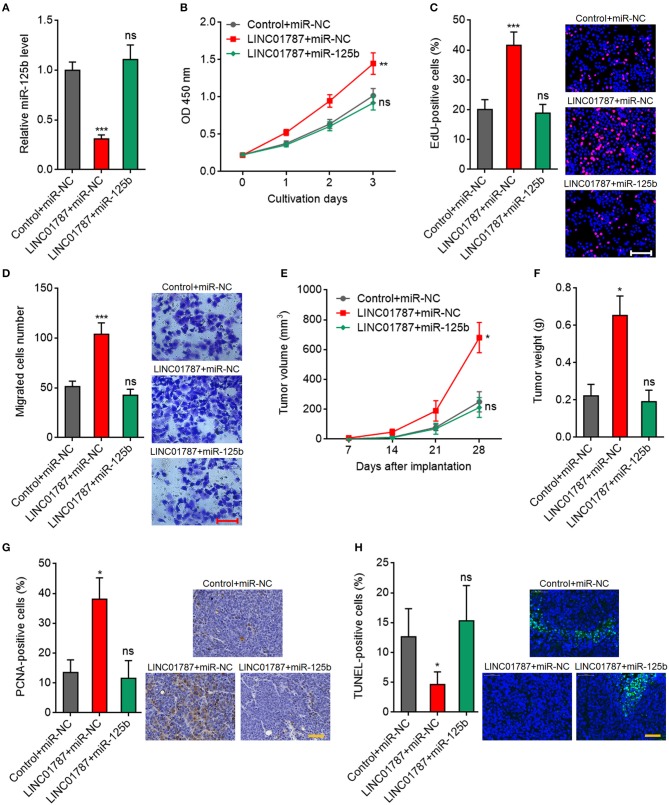
miR-125b overexpression reverses the oncogenic roles of LINC01787 in breast cancer. **(A)** miR-125b expression in LINC01787 and miR-125b doubly stably overexpressed and control MDA-MB-231 cells was detected by qRT-PCR. **(B)** Cell proliferation of LINC01787 and miR-125b doubly stably overexpressed and control MDA-MB-231 cells was detected by CCK-8 assays. **(C)** Cell proliferation of LINC01787 and miR-125b doubly stably overexpressed and control MDA-MB-231 cells was detected by EdU incorporation assays. Red colors indicate EdU-positive and proliferative cells. Scale bar = 100 μm. **(D)** Cell migration of LINC01787 and miR-125b doubly stably overexpressed and control MDA-MB-231 cells was detected by transwell assays. Scale bar = 100 μm. For **(A–D)**, results are shown as mean ± SD of 3 independent experiments. ^**^P < 0.01, ^***^P < 0.001, ns, not significant by one-way ANOVA followed by Dunnett's multiple comparisons test, compared with control+miR-NC group. **(E)** LINC01787 and miR-125b doubly stably overexpressed and control MDA-MB-231 cells were subcutaneously implanted into nude mice. Tumor volume was detected every 7 days. **(F)** Subcutaneous tumor weight was detected at the 28th day after implantation. **(G)** *in vivo* cell proliferation of LINC01787 and miR-125b doubly stably overexpressed and control MDA-MB-231 cells was detected by PCNA IHC staining. Scale bar = 50 μm. **(H)** *in vivo* cell apoptosis of LINC01787 and miR-125b doubly stably overexpressed and control MDA-MB-231 cells was detected by TUNEL staining. Scale bar = 50 μm. For **(E–H)**, results are shown as mean ± SD of 5 mice in each group. ^*^P < 0.05, ns, not significant by Kruskal–Wallis test followed by Dunn's multiple comparisons test, compared with control+miR-NC group.

To further elucidate whether the oncogenic roles of LINC01787 in breast cancer are dependent on the negative regulation of miR-125b, we stably overexpressed miR-125b in LINC01787 stably overexpressed MDA-MB-231 cells (Figure 6A). CCK-8 assays showed that enhanced expression of miR-125b reversed the accelerated cell proliferation caused by LINC01787 overexpression (Figure 6B). EdU incorporation assays also revealed that enhanced expression of miR-125b reversed the pro-proliferative roles of LINC01787 in breast cancer (Figure 6C). Transwell assays showed that enhanced expression of miR-125b reversed the increased migration ability of breast cancer cells caused by LINC01787 overexpression (Figure 6D). These data suggested that miR-125b overexpression reverses the roles of LINC01787 in promoting breast cancer cell proliferation and migration. In addition, miR-125b and LINC01787 stably overexpressed and control MDA-MB-231 cells were subcutaneously implanted into nude mice. The results showed that enhanced expression of miR-125b reversed the accelerated xenograft growth caused by LINC01787 overexpression (Figures 6E,F). LINC01787 and miR-125b overexpression efficiencies were confirmed in the xenografts (Figure S2A). IHC detection of miR-125b targets KIAA1522, ETS1, and SNAI1 in the xenografts revealed that LINC01787 overexpression up-regulated the protein levels of KIAA1522, ETS1, and SNAI1, which were abolished by the overexpression of miR-125b (Figures S2B–D). Proliferation marker PCNA IHC staining showed that enhanced expression of miR-125b reversed the increasing of PCNA positive cells caused by LINC01787 overexpression (Figure 6G). TUNEL assays showed that enhanced expression of miR-125b reversed the reduction of apoptotic cells caused by LINC01787 overexpression (Figure 6H). Collectively, these data suggested that miR-125b overexpression reverses the oncogenic roles of LINC01787 in breast cancer.

### LINC01787 Is Up-Regulated in Breast Cancer and Associated With Poor Prognosis

**Figure 7:**
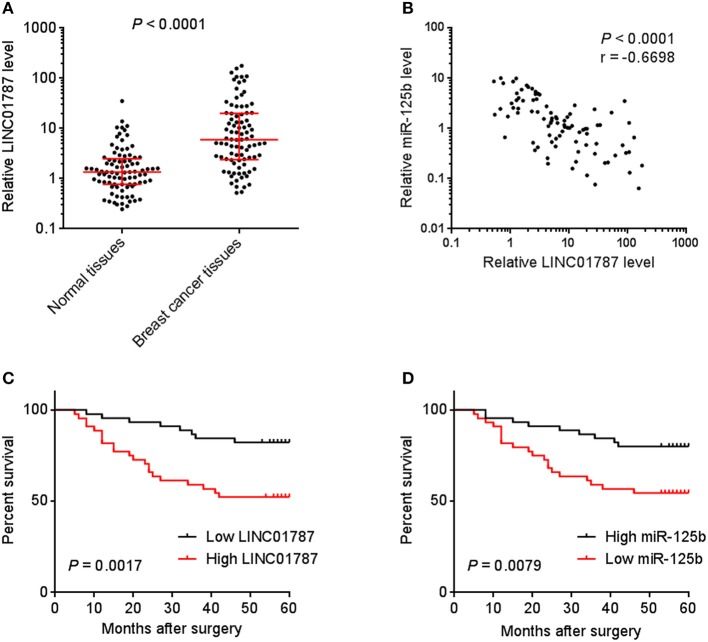
The correlation between LINC01787 expression, miR-125b expression, and prognosis of breast cancer patients. **(A)** LINC01787 expression in 89 pairs of breast cancer tissues and normal adjacent tissues was measured by qRT-PCR. *P* < 0.0001 by Wilcoxon signed rank test, compared with normal adjacent tissues group. **(B)** The correlation between LINC01787 expression level and miR-125b expression level in 89 breast cancer tissues. *r* = −0.6698, *P* < 0.0001 by Spearman correlation analysis. **(C)** Kaplan–Meier analyses of the correlations between LINC01787 expression level and overall survival of *n* = 89 breast cancer patients. *P* = 0.0017 by Log-rank test. **(D)** Kaplan–Meier analyses of the correlations between miR-125b expression level and overall survival of *n* = 89 breast cancer patients. *P* = 0.0079 by Log-rank test.

To investigate the clinical significance of LINC01787 in breast cancer, we collected 89 pairs of breast cancer tissues and normal adjacent tissues. The expression of LINC01787 in these tissues was measured by qRT-PCR. The results showed that LINC01787 was significantly up-regulated in breast cancer tissues compared with normal adjacent tissues (Figure 7A). The expression of miR-125b was measured in the same breast cancer tissues. The results showed that miR-125b expression level was inversely associated with LINC01787 expression level in breast cancer tissues (Figure 7B). Analyzing the correlation between LINC01787 expression level and clinicopathologic characteristics of these 89 breast cancer patients revealed that high expression level of LINC01787 was positively correlated with tumor size, lymph node metastasis, and advanced clinical stage (Table 1). No correlation was observed between LINC01787 expression level and age, ER status, PR status, or CerbB2 status (Table 1). The correlation between LINC01787 expression level and prognosis of these 89 breast cancer patients showed that breast cancer patients with high LINC01787 expression level had worse prognosis that those with low LINC01787 expression level (Figure 7C). The correlation between LINC01787 expression levels and prognosis of breast cancer patients was further analyzed by The Kaplan Meier plotter (http://kmplot.com/analysis/), which includes 1089 breast cancer cases. The result also showed that high expression of LINC01787 was associated with poor prognosis of breast cancer patients (Figure S3). Consistent with previous report (24), breast cancer patients with low miR-125 expression level also had worse prognosis that those with high miR-125 expression level in our cohort (Figure 7D). These data support the negative regulation of miR-125b by LINC01787 *in vivo*. These data also demonstrated that LINC01787 is up-regulated in breast cancer and associated with poor prognosis of breast cancer patients.

**Table 1:** Relationship between LINC01787 expression level and clinicopathologic characteristics of breast cancer.

**Parameters**	**LINC01787**		***P*-value**
	**Low**	**High**	
Age			0.913
>50	22	21	
≤50	23	23	
Tumor size (cm)			0.025
≤2 cm	20	11	
2–5 cm	21	20	
>5 cm	4	13	
Lymph node status			0.010
N0–N1	36	24	
N2–N3	9	20	
Clinical stage			0.013
I	18	7	
II	17	16	
III	10	21	
ER status			0.753
Negative	24	22	
Positive	21	22	
PR status			0.736
Negative	20	18	
Positive	25	26	
CerbB2 status			0.112
0, 1+	29	21	
2+, 3+	16	23	

*LINC01787 median expression level was used as the cutoff. P-value was calculated by Pearson chi-square test*.

## Discussion

More and more studies are focusing on the important roles and clinical significance of lncRNAs in cancers (43). Due to their key roles in cancers, enhancing tumor suppressive lncRNAs or targeting oncogenic lncRNAs represent novel therapeutic approaches for various cancers. The aberrant expressions and important roles of lncRNAs in breast cancer have begun to be revealed (44–46). lncRNA CYTOR (LINC00152) was revealed to be up-regulated in breast cancer and associated with poor survival of breast cancer patients (44). CYTOR was also shown to be required for breast cancer cell proliferation, migration, and cytoskeletal organization (44). lncRNA DSCAM-AS1 was found to promote breast cancer progression and tamoxifen resistance (45). LncRNA-Hh was revealed to enhance breast cancer stem cells generation (46). Although several lncRNAs have been studied in breast cancer, the roles of lncRNAs in breast cancer are far from being understood.

In this study, we identified a novel lncRNA LINC01787, which locates at chromosome 1p21.3 and has six exons containing 1994 nucleotides in length. The expression and roles of LINC01787 in cancers are still unknown. In this study, we found that LINC01787 is up-regulated in breast cancer and associated with larger tumor size, lymph node metastasis, advanced clinical stage and poor prognosis of breast cancer patients. Functional assays showed that overexpression of LINC01787 enhances the proliferation and migration of breast cancer cells, and conversely knockdown of LINC01787 represses the proliferation and migration of breast cancer cells. Furthermore, we also found that LINC01787 promotes breast cancer xenograft growth *in vivo*. Thus, we identified that LINC01787 plays key oncogenic roles in breast cancer. Our data also suggested that LINC01787 is a potential therapeutic target for breast cancer.

The molecular mechanisms underlying the roles of lncRNAs are complex. The most frequently reported mechanism is to bind proteins and change the location, modification, expression, and function of the interacted proteins (47). Another common mechanism of lncRNAs is to competitively bind common miRNAs and relieve the repressive roles of miRNAs on their targets (48). These lncRNAs are classified as competing endogenous RNA (ceRNA), such as the binding of miR-200s by lncRNA-ATB, the binding of miR-133 and miR-135 by linc-MD1, and the binding of miR-145 and miR-181a by lincRNA-RoR (49–51). In this study, we identified a relative novel mechanism of lncRNA. We found that LINC01787 could specifically bind pre-miR-125b. Mature miR-125b, also known as miR-125b-5p, was generated from two genomic sites. *MIR125B1* is located at chromosome 11q24.1, which transcribes pre-miR-125b-1. *MIR125B2* is located at chromosome 21q21.1, which transcribes pre-miR-125b-2. Both pre-miR-125b-1 and pre-miR-125b-2 are bound and processed by DICER to generate mature miR-125b. In this study, using RNA pull-down assay with *in vitro*-transcribed LINC01787 and ChIRP assay with LINC01787 antisense biotinylated probes, we found that LINC01787 specifically binds both pre-miR-125b. Although both these assays could not exclude the potential that the interaction between LINC01787 and pre-miR-125b might be mediated by other factors, such as proteins, the findings that the mutation of pre-miR-125b binding sites on LINC01787 abolished the interaction between LINC01787 and pre-miR-125b are in favor of a direct interaction between LINC01787 and pre-miR-125b. Further, we found that via binding pre-miR-125b, LINC01787 represses the binding between pre-miR-125b and DICER, represses the processing of pre-miR-125b by DICER, and therefore inducing the accumulation of pre-miR-125b and repressing mature miR-125b generation. Through inhibiting mature miR-125 generation, LINC01787 up-regulates the expression of miR-125b targets including KIAA1522, ETS1, and SNAI1. Therefore, we provided new evidence about the regulation of miRNA by lncRNA via modulating miRNAs generation.

The negative regulation of miR-125b by LINC01787 was further verified by functional rescue assays and expression association in clinical tissues. The mutation of the pre-miR-125b binding sites on LINC01787 abolished the roles of LINC01787 in promoting breast cancer cell proliferation, migration, and *in vivo* xenograft growth. Furthermore, overexpression of miR-125b also abolished the roles of LINC01787 in promoting breast cancer cell proliferation, migration, and *in vivo* xenograft growth. Thus, these functional rescue assays suggested that the oncogenic roles of LINC01787 are dependent on the negative modulation of miR-125b. The expression correlation of LINC01787 and miR-125b was analyzed in 89 breast cancer tissues. Our data revealed that the expression of miR-125b was significantly inversely correlated with that of LINC01787 in breast cancer tissues. Conversely to LINC01787, low expression of miR-125b predicts poor survival of breast cancer patients. The inverse expression association between LINC01787 and miR-125b supports the negative regulation of miR-125b by LINC01787 *in vivo*.

In conclusion, our results identified LINC01787 as a critical oncogenic lncRNA in breast cancer, promoting breast cancer cell proliferation, migration, and *in vivo* xenograft growth via binding pre-miR-125b and repressing mature miR-125b generation. Furthermore, we identified LINC01787 as an up-regulated and poor survival-associated lncRNA in breast cancer. Our results thus suggests that LINC01787 is a potential prognostic biomarker and therapeutic target for breast cancer.

## Data Availability Statement

The raw data supporting the conclusions of this manuscript will be made available by the authors, without undue reservation, to any qualified researcher.

## Ethics Statement

The studies involving human participants were reviewed and approved by the Ethics Committee of Xinxiang Medical University (Xinxiang, China). The patients/participants provided their written informed consent to participate in this study. The animal study was reviewed and approved by the Ethics Committee of Xinxiang Medical University (Xinxiang, China).

## Author Contributions

YW and YL designed this study, analyzed the data, and wrote the paper. YL, YS, ZW, ZZ, and ML performed the experiments. All authors have read and approved the final manuscript and agree to be accountable for the content of the work.

### Conflict of Interest

The authors declare that the research was conducted in the absence of any commercial or financial relationships that could be construed as a potential conflict of interest.

## References

[1] Bray F Ferlay J Soerjomataram I Siegel RL Torre LA Jemal A. Global cancer statistics 2018: GLOBOCAN estimates of incidence and mortality worldwide for 36 cancers in 185 countries. CA Cancer J Clin. (2018) 68:394–424. 10.3322/caac.21492 30207593

[2] Shen M Jiang YZ Wei Y Ell B Sheng X Esposito M. Tinagl1 suppresses triple-negative breast cancer progression and metastasis by simultaneously inhibiting integrin/FAK and EGFR signaling. Cancer Cell. (2019) 35:64–80.e7. 10.1016/j.ccell.2018.11.016 30612941

[3] Ishay-Ronen D Diepenbruck M Kalathur RKR Sugiyama N Tiede S Ivanek R. Gain fat-lose metastasis: converting invasive breast cancer cells into adipocytes inhibits cancer metastasis. Cancer Cell. (2019) 35:17–32.e6. 10.1016/j.ccell.2018.12.002 30645973

[4] Salatino M Girotti MR Rabinovich GA. Glycans pave the way for immunotherapy in triple-negative breast cancer. Cancer Cell. (2018) 33:155–7. 10.1016/j.ccell.2018.01.015 29438689

[5] Keklikoglou I Cianciaruso C Guc E Squadrito ML Spring LM Tazzyman S. Chemotherapy elicits pro-metastatic extracellular vesicles in breast cancer models. Nat Cell Biol. (2019) 21:190–202. 10.1038/s41556-018-0256-3 30598531 PMC6525097

[6] Rios AC Capaldo BD Vaillant F Pal B van Ineveld R Dawson CA Intraclonal plasticity in mammary tumors revealed through large-scale single-cell resolution 3D imaging. Cancer Cell. (2019) 35:618–32.e6. 10.1016/j.ccell.2019.02.010 30930118

[7] Jiang YZ Ma D Suo C Shi J Xue M Hu X. Genomic and transcriptomic landscape of triple-negative breast cancers: subtypes and treatment strategies. Cancer Cell. (2019) 35:428–40.e5. 10.1016/j.ccell.2019.02.001 30853353

[8] Cassetta L Fragkogianni S Sims AH Swierczak A Forrester LM Zhang H. Human tumor-associated macrophage and monocyte transcriptional landscapes reveal cancer-specific reprogramming, biomarkers, and therapeutic targets. Cancer Cell. (2019) 35:588–602.e10. 10.1016/j.ccell.2019.02.009 30930117 PMC6472943

[9] Carlson P Dasgupta A Grzelak CA Kim J Barrett A Coleman IM. Targeting the perivascular niche sensitizes disseminated tumour cells to chemotherapy. Nat Cell Biol. (2019) 21:238–50. 10.1038/s41556-018-0267-0 30664790 PMC6948102

[10] Iyer MK Niknafs YS Malik R Singhal U Sahu A Hosono Y. The landscape of long noncoding RNAs in the human transcriptome. Nat Genet. (2015) 47:199–208. 10.1038/ng.3192 25599403 PMC4417758

[11] Esposito R Bosch N Lanzos A Polidori T Pulido-Quetglas C Johnson R. Hacking the cancer genome: profiling therapeutically actionable long non-coding RNAs using CRISPR-Cas9 screening. Cancer Cell. (2019) 35:545–57. 10.1016/j.ccell.2019.01.019 30827888

[12] Yao RW Wang Y Chen L. Cellular functions of long noncoding RNAs. Nat Cell Biol. (2019) 21:542–51. 10.1038/s41556-019-0311-8 31048766

[13] Inui M Mokuda S Sato T Tamano M Takada S Asahara H. Dissecting the roles of miR-140 and its host gene. Nat Cell Biol. (2018) 20:516–8. 10.1038/s41556-018-0077-4 29695789 PMC6373877

[14] Zhang L Dong Y Wang Y Gao J Lv J Sun J. Long non-coding RNAs in ocular diseases: new and potential therapeutic targets. FEBS J. (2019) 286:2261–72. 10.1111/febs.14827 30927500

[15] Rupaimoole R Slack FJ. MicroRNA therapeutics: towards a new era for the management of cancer and other diseases. Nat Rev Drug Discov. (2017) 16:203–22. 10.1038/nrd.2016.246 28209991

[16] Jahagirdar D Purohit S Jain A Sharma NK. Export of microRNAs: a Bridge between breast carcinoma and their neighboring cells. Front Oncol. (2016) 6:147. 10.3389/fonc.2016.00147 27379209 PMC4913210

[17] Mittag T Fawzi NL. Protein quality and miRNA slicing get into phase. Nat Cell Biol. (2018) 20:635–7. 10.1038/s41556-018-0113-4 29802409

[18] Yuan JH Yang F Chen BF Lu Z Huo XS Zhou WP. The histone deacetylase 4/SP1/microrna-200a regulatory network contributes to aberrant histone acetylation in hepatocellular carcinoma. Hepatology. (2011) 54:2025–35. 10.1002/hep.24606 21837748

[19] Piras S Furfaro AL Caggiano R Brondolo L Garibaldi S Ivaldo C. microRNA-494 favors HO-1 expression in neuroblastoma cells exposed to oxidative stress in a bach1-independent way. Front Oncol. (2018) 8:199. 10.3389/fonc.2018.00199 29951371 PMC6008388

[20] Bu Y Yoshida A Chitnis N Altman BJ Tameire F Oran A. A PERK-miR-211 axis suppresses circadian regulators and protein synthesis to promote cancer cell survival. Nat Cell Biol. (2018) 20:104–15. 10.1038/s41556-017-0006-y 29230015 PMC5741512

[21] Clancy JW Zhang Y Sheehan C D'Souza-Schorey C. An ARF6-Exportin-5 axis delivers pre-miRNA cargo to tumour microvesicles. Nat Cell Biol. (2019) 21:856–66. 10.1038/s41556-019-0345-y 31235936 PMC6697424

[22] Zhang L Yang F Yuan JH Yuan SX Zhou WP Huo XS. Epigenetic activation of the MiR-200 family contributes to H19-mediated metastasis suppression in hepatocellular carcinoma. Carcinogenesis. (2013) 34:577–86. 10.1093/carcin/bgs381 23222811

[23] Li Y Wang Y Fan H Zhang Z Li N. miR-125b-5p inhibits breast cancer cell proliferation, migration and invasion by targeting KIAA1522. Biochem Biophys Res Commun. (2018) 504:277–82. 10.1016/j.bbrc.2018.08.172 30177391

[24] Zhang Y Yan LX Wu QN Du ZM Chen J Liao DZ. miR-125b is methylated and functions as a tumor suppressor by regulating the ETS1 proto-oncogene in human invasive breast cancer. Cancer Res. (2011) 71:3552–62. 10.1158/0008-5472.CAN-10-2435 21444677

[25] Dong H Hu J Zou K Ye M Chen Y Wu C. Activation of LncRNA TINCR by H3K27 acetylation promotes Trastuzumab resistance and epithelial-mesenchymal transition by targeting MicroRNA-125b in breast Cancer. Mol Cancer. (2019) 18:3. 10.1186/s12943-018-0931-9 30621694 PMC6323810

[26] Ponting CP Oliver PL Reik W. Evolution and functions of long noncoding RNAs. Cell. (2009) 136:629–41. 10.1016/j.cell.2009.02.006 19239885

[27] Yan X Hu Z Feng Y Hu X Yuan J Zhao SD. Comprehensive genomic characterization of long non-coding RNAs across human cancers. Cancer Cell. (2015) 28:529–40. 10.1016/j.ccell.2015.09.006 26461095 PMC4777353

[28] Berger AC Korkut A Kanchi RS Hegde AM Lenoir W Liu W. A Comprehensive pan-cancer molecular study of gynecologic and breast cancers. Cancer Cell. (2018) 33:690–705.e9. 10.1016/j.ccell.2018.03.014 29622464 PMC5959730

[29] Wang Z Yang B Zhang M Guo W Wu Z Wang Y. lncRNA epigenetic landscape analysis identifies EPIC1 as an oncogenic lncRNA that interacts with MYC and promotes cell-cycle progression in cancer. Cancer Cell. (2018) 33:706–20.e9. 10.1016/j.ccell.2018.03.006 29622465 PMC6143179

[30] Mondal T Juvvuna PK Kirkeby A Mitra S Kosalai ST Traxler L. Sense-antisense lncRNA pair encoded by locus 6p22.3 Determines neuroblastoma susceptibility via the USP36-CHD7-SOX9 regulatory axis. Cancer Cell. (2018) 33:417–34.e7. 10.1016/j.ccell.2018.01.020 29533783

[31] Yuan JH Liu XN Wang T Pan W Tao QF Zhou WP. The MBNL3 splicing factor promotes hepatocellular carcinoma by increasing PXN expression through the alternative splicing of lncRNA-PXN-AS1. Nat Cell Biol. (2017) 19:820–32. 10.1038/ncb3538 28553938

[32] Chen F Chen J Yang L Liu J Zhang X Zhang Y. Extracellular vesicle-packaged HIF-1α-stabilizing lncRNA from tumour-associated macrophages regulates aerobic glycolysis of breast cancer cells. Nat Cell Biol. (2019) 21:498–510. 10.1038/s41556-019-0299-0 30936474

[33] Li JK Chen C Liu JY Shi JZ Liu SP Liu B. Long noncoding RNA MRCCAT1 promotes metastasis of clear cell renal cell carcinoma via inhibiting NPR3 and activating p38-MAPK signaling. Mol Cancer. (2017) 16:111. 10.1186/s12943-017-0681-0 28659173 PMC5490088

[34] Zhang C Yuan J Hu H Chen W Liu M Zhang J. Long non-coding RNA CHCHD4P4 promotes epithelial-mesenchymal transition and inhibits cell proliferation in calcium oxalate-induced kidney damage. Braz J Med Biol Res. (2017) 51:e6536. 10.1590/1414-431x20176536 29160413 PMC5685061

[35] Derderian C Orunmuyi AT Olapade-Olaopa EO Ogunwobi O. PVT1 Signaling is a mediator of cancer progression. Front Oncol. (2019) 9:502. 10.3389/fonc.2019.00502 31249809 PMC6582247

[36] Zheng D Zhang Y Hu Y Guan J Xu L Xiao W. Long noncoding RNA Crnde attenuates cardiac fibrosis via Smad3-Crnde negative feedback in diabetic cardiomyopathy. FEBS J. (2019) 286:1645–55. 10.1111/febs.14780 30748104 PMC6849551

[37] Hu WL Jin L Xu A Wang YF Thorne RF Zhang XD. GUARDIN is a p53-responsive long non-coding RNA that is essential for genomic stability. Nat Cell Biol. (2018) 20:492–502. 10.1038/s41556-018-0066-7 29593331

[38] Zhu XT Yuan JH Zhu T Li Y Cheng XY. Long noncoding RNA glypican 3 (GPC3) antisense transcript 1 promotes hepatocellular carcinoma progression via epigenetically activating GPC3. FEBS J. (2016) 283:3739–54. 10.1111/febs.13839 27573079

[39] Wang H Wang X Li X Wang Q Qing S Zhang Y. A novel long non-coding RNA regulates the immune response in MAC-T cells and contributes to bovine mastitis. FEBS J. (2019) 286:1780–95. 10.1111/febs.14783 30771271

[40] Wang CJ Zhu C Xu J Wang M Zhao WY Liu Q The lncRNA UCA1 promotes proliferation, migration, immune escape and inhibits apoptosis in gastric cancer by sponging anti-tumor miRNAs. Mol Cancer. (2019) 18:115 10.1186/s12943-019-1059-2 31272462 PMC6609402

[41] Zhang G Li S Lu J Ge Y Wang Q Ma G. LncRNA MT1JP functions as a ceRNA in regulating FBXW7 through competitively binding to miR-92a-3p in gastric cancer. Mol Cancer. (2018) 17:87. 10.1186/s12943-018-0829-6 29720189 PMC5930724

[42] Tian T Lv X Pan G Lu Y Chen W He W. Long noncoding RNA MPRL promotes mitochondrial fission and cisplatin chemosensitivity via disruption of pre-miRNA processing. Clin Cancer Res. (2019) 25:3673–88. 10.1158/1078-0432.CCR-18-2739 30885939 PMC8725174

[43] Keshavarz M Asadi MH. Long non-coding RNA ES1 controls the proliferation of breast cancer cells by regulating the Oct4/Sox2/miR-302 axis. FEBS J. (2019) 286:2611–23. 10.1111/febs.14825 30927330

[44] Van Grembergen O Bizet M de Bony EJ Calonne E Putmans P Brohee S. Portraying breast cancers with long noncoding RNAs. Sci Adv. (2016) 2:e1600220. 10.1126/sciadv.1600220 27617288 PMC5010371

[45] Niknafs YS Han S Ma T Speers C Zhang C Wilder-Romans K. The lncRNA landscape of breast cancer reveals a role for DSCAM-AS1 in breast cancer progression. Nat Commun. (2016) 7:12791. 10.1038/ncomms12791 27666543 PMC5052669

[46] Zhou M Hou Y Yang G Zhang H Tu G Du YE. LncRNA-Hh strengthen cancer stem cells generation in twist-positive breast cancer via activation of hedgehog signaling pathway. Stem Cells. (2016) 34:55–66. 10.1002/stem.2219 26418365 PMC4832137

[47] Xu D Yang F Yuan JH Zhang L Bi HS Zhou C. Long noncoding RNAs associated with liver regeneration 1 accelerates hepatocyte proliferation during liver regeneration by activating Wnt/β -catenin signaling. Hepatology. (2013) 58:739–51. 10.1002/hep.26361 23483581

[48] Salmena L Poliseno L Tay Y Kats L Pandolfi P. A ceRNA hypothesis: the Rosetta Stone of a hidden RNA language? Cell. (2011) 146:353–8. 10.1016/j.cell.2011.07.014 21802130 PMC3235919

[49] Yuan JH Yang F Wang F Ma JZ Guo YJ Tao QF. A long noncoding RNA activated by TGF-β promotes the invasion-metastasis cascade in hepatocellular carcinoma. Cancer Cell. (2014) 25:666–81. 10.1016/j.ccr.2014.03.010 24768205

[50] Cesana M Cacchiarelli D Legnini I Santini T Sthandier O Chinappi M. A long noncoding RNA controls muscle differentiation by functioning as a competing endogenous RNA. Cell. (2011) 147:358–69. 10.1016/j.cell.2011.09.028 22000014 PMC3234495

[51] Wang Y Xu Z Jiang J Xu C Kang J Xiao L. Endogenous miRNA sponge lincRNA-RoR regulates Oct4, Nanog, and Sox2 in human embryonic stem cell self-renewal. Dev Cell. (2013) 25:69–80. 10.1016/j.devcel.2013.03.002 23541921

