# Supplementary material for: Long Non-coding RNA LINC01787 Drives Breast Cancer Progression via Disrupting miR-125b Generation
Source: Front Oncol. 2019 Nov 5;9:1140. doi: 10.3389/fonc.2019.01140 (PMC6848230; doi:10.3389/fonc.2019.01140)
Supplement: Supplementary file 1 [file Data_Sheet_1.pdf]

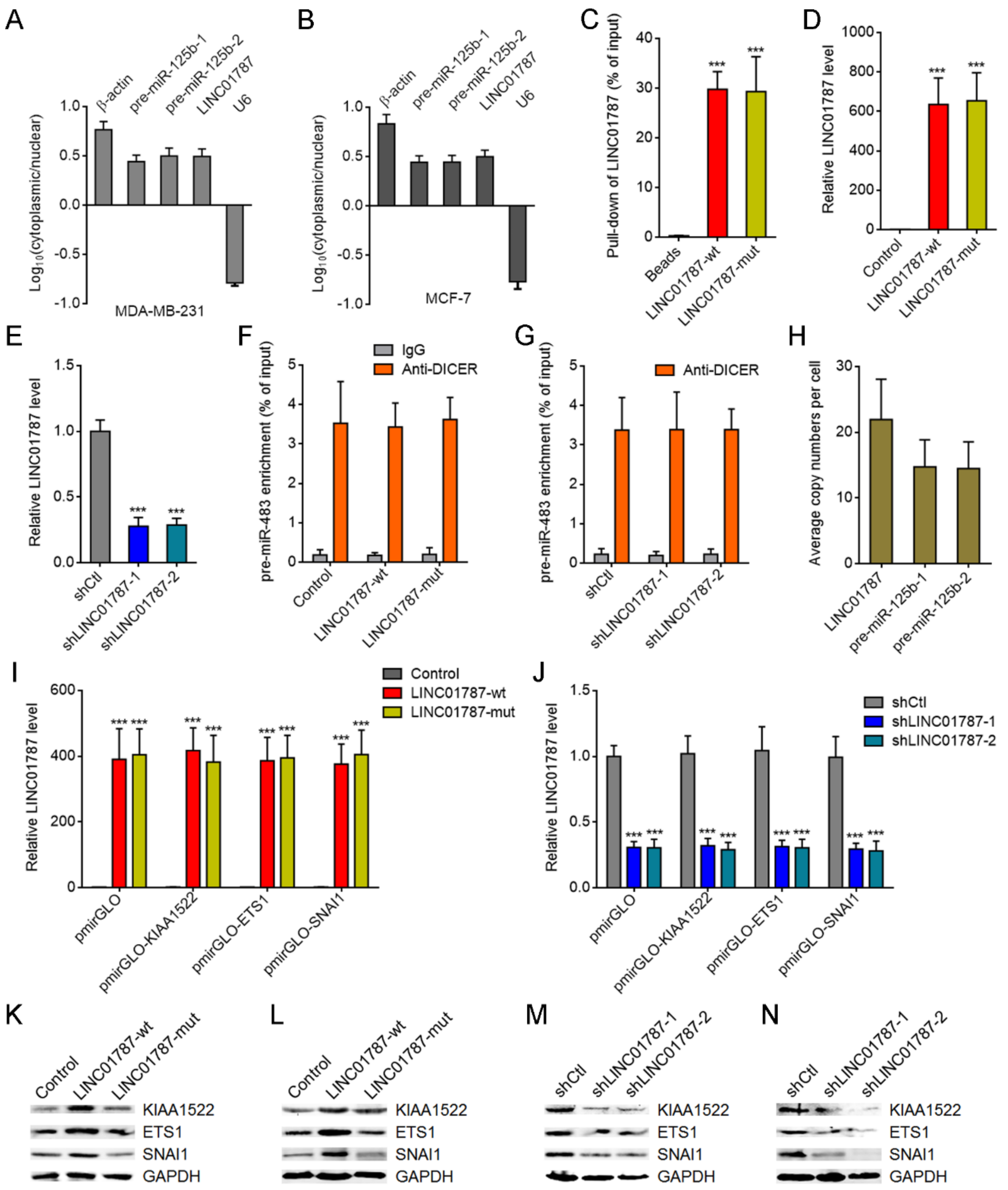

Figure S1. LINC01787 binds pre-miR-125b and represses miR-125b generation. (**A**,**B**) The levels of pre-miR-125b-1, pre-miR-125b-2, and LINC01787 in purified cytoplasmic and nuclear RNAs from MDA-MB-231 (**A**) or MCF-7 (**B**) cells. β-actin and U6 serve as cytoplasmic and nuclear control, respectively. (**C**) RNA pull-down assays using in vitro transcribed biotin-labelled wild type LINC01787 (LINC01787-wt) or pre-miR-125b binding sites mutated LINC01787 (LINC01787-mut). The efficiencies of LINC01787-wt and LINC01787-mut recovery were detected. (**D**) After transient overexpression of LINC01787-wt or LINC01787-mut in MDA-MB-231 cells, LINC01787 expression was confirmed by qRT-PCR. (**E**) After transient silencing of

LINC01787 in MDA-MB-231 cells, LINC01787 expression was confirmed by qRT-PCR. (**F**) After transient overexpression of LINC01787-wt or LINC01787-mut in MDA-MB-231 cells, RIP assays were performed to detect the binding between DICER and pre-miR-483. (**G**) After transient silencing of LINC01787 in MDA-MB-231 cells, RIP assays were performed to detect the binding between DICER and pre-miR-483. (**H**) The exact molecular numbers of LINC01787, pre-miR-125b-1, and pre-miR-125b-2 were detected by quantitative PCR. (**I**) LINC01787 expression in MCF-7 cells co-transfected with luciferase reporters containing nothing, 3'-UTR of KIAA1522, 3'-UTR of ETS1, or 3'-UTR of SNAI1 and LINC01787-wt or LINC01787-mut overexpression plasmids. (**J**) LINC01787 expression in MDA-MB-231 cells co-transfected with luciferase reporters containing nothing, 3'-UTR of KIAA1522, 3'-UTR of ETS1, or 3'-UTR of SNAI1 and LINC01787 specific shRNAs. (**K**,**L**) After transient overexpression of LINC01787-wt or LINC01787-mut in MCF-7 cells, the expression of miR-125b targets KIAA1522, ETS1, and SNAI1 was measured by western blot. (**M**,**N**) After transient silencing of LINC01787 in MDA-MB-231 cells, the expression of miR-125b targets KIAA1522, ETS1, and SNAI1 was measured by western blot. Results are shown as mean ± SD of 3 independent experiments. ***P <

0.001 by one-way ANOVA followed by Dunnett's multiple comparisons test, compared with beads, control, or shCtl group.

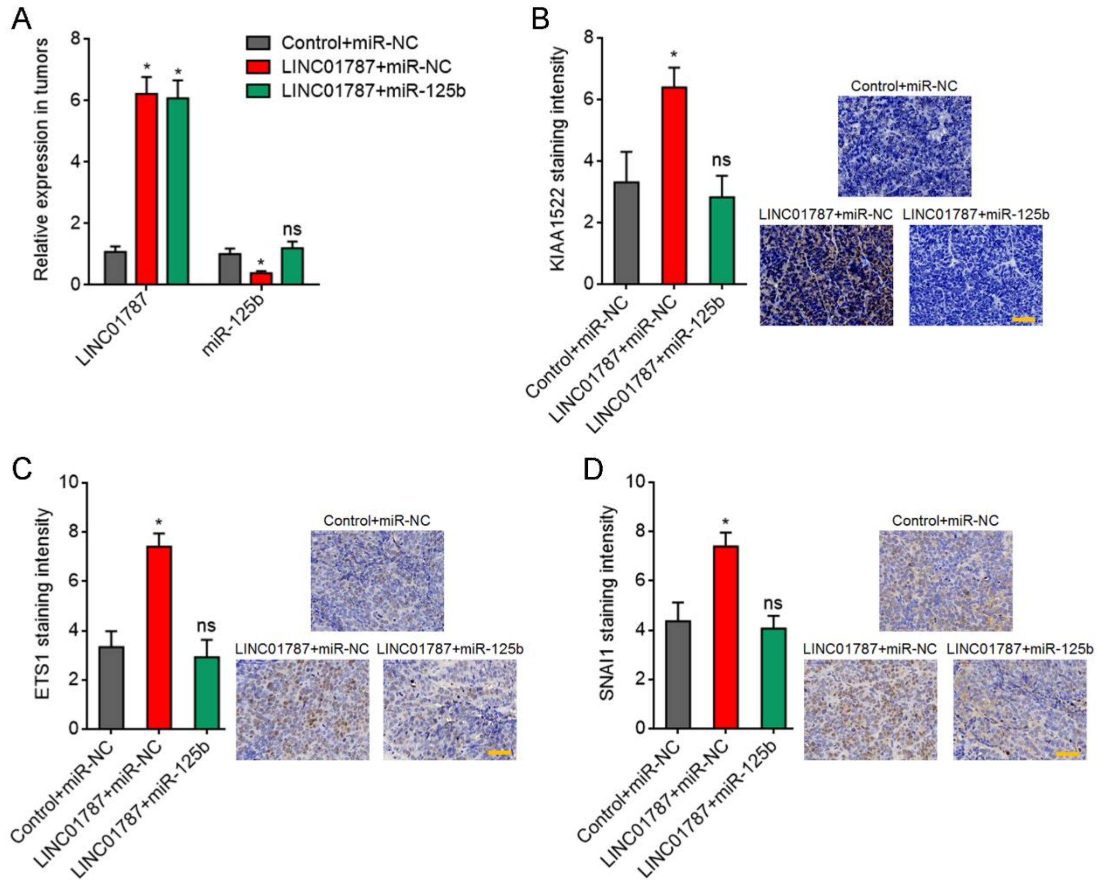

Figure S2. LINC01787 upregulates the expression of miR-125b targets in vivo. (**A**) LINC01787 and miR-125b doubly stably overexpressed and control MDA-MB-231 cells were subcutaneously implanted into nude mice. LINC01787 and miR-125b expression in the xenografts was detected by qRT-PCR. (**B**-**D**) miR-125b targets KIAA1522 (**B**), ETS1 (**C**), and SNAI1 (**D**) expression levels in the xenografts were detected by IHC staining. Scale bar = 50 μm. Results are shown as mean ± SD of 5

mice in each group. *$P < 0.05$, ns, not significant by Kruskal-Wallis test followed by Dunn's multiple comparisons test, compared with control+miR-NC group.

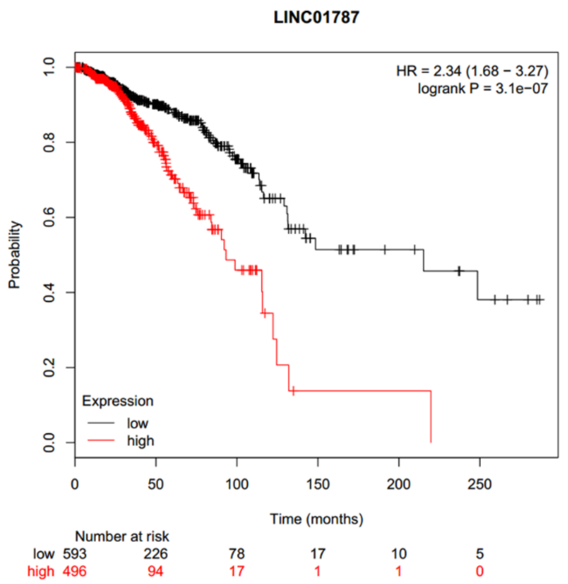

Figure S3. The correlation between LINC01787 expression levels and prognosis of breast cancer patients was analysed by The Kaplan Meier plotter (http://kmplot.com/analysis/), which includes 1089 breast cancer cases.
